# Tonic-Clonic Seizure in Patient With SLE: Posterior Reversible Encephalopathy Syndrome, or a Neuropsychiatric Manifestation of SLE?

**DOI:** 10.31138/mjr.20230905.tc

**Published:** 2023-09-05

**Authors:** Yasin Ozturk, Neslihan Ozturk, Aysenur Argun, Hakan Ozer, Fethi Yonet, İsmail Baloglu

**Affiliations:** 1 Nephrology Department, Meram School of Medicine, Necmettin Erbakan University, Turkey,; 2 Internal Medicine Department, Meram School of Medicine, Necmettin Erbakan University, Turkey

**Keywords:** Systemic Lupus Erythematosus, PRES, neuropsychiatric SLE, MRI

## Abstract

Posterior reversible encephalopathy syndrome (PRES) is a clinically and radiologically diagnosed reversible sudden onset disease with many neurological symptoms. SLE is the most common cause of PRES among autoimmune diseases. Many factors, such as SLE activity, hypertension, hematological and renal diseases, lymphopenia dyslipidemia, and immunosuppressive treatments, can trigger PRES in SLE. We wanted to draw attention to the difference between neuropsychiatric systemic lupus erythematosus (SLE) and PRES in a patient with SLE and the triggers for developing PRES in SLE by presenting a hypertensive patient on immunosuppressive therapy who had just started haemodialysis treatment and had generalised tonic-clonic seizures.

## INTRODUCTION

Posterior reversible encephalopathy syndrome (PRES) is a neurological disorder with an acute or subacute onset of unknown incidence, characterised by various symptoms that may include headache, visual disturbances, consciousness disorders, seizures, and focal neurological deficits. Different mechanisms contribute to the development of PRES. Initially, hypertension causes hyperperfusion due to impaired cerebrovascular auto-regulation. On the other hand, hyperperfusion causes the blood-brain barrier’s destruction, causing plasma and macromolecules to escape into the interstitial space via tight junction proteins. The release of cytokines activates the secretion of vasoactive factors from endothelial cells, which increases vascular permeability, leading to interstitial oedema. Because of these changes in endothelial function, vasogenic oedema occurs in the central nervous system circulation. It is seen primarily in the parieto-occipital region on cranial imaging.^1^ PRES is often caused by cytotoxic drugs, (pre)eclampsia, sepsis, kidney disease, immunosuppression, or autoimmune disorders. Systemic Lupus Erythematosus (SLE) accompanies a significant portion of the admitted cases with PRES.^2^ The treatment is symptomatic and is determined according to the underlying condition. The overall prognosis is favorable, as most patients’ clinical symptoms and imaging findings are reversible. However, neurologic sequelae, including long-term epilepsy, may persist in individual cases.^3^

Risk factors for PRES in SLE include hypertension, renal dysfunction, cytopenia, dyslipidemia, heart failure, high SLE activity index scores, and young age. The prevalence of PRES in patients with SLE ranges from 0.7% to 1.4% and recurs in 13% of cases.^4^ When neurological symptoms, especially seizures, develop in patients with SLE, PRES, and neuropsychiatric SLE should be differentiated. In addition, the literature has argued that PRES may be a part of Neuropsychiatric Lupus (NPSLE) syndromes.^1^ While tonic-clonic seizures are seen at a rate of 85% in PRES, they can be detected at rates ranging from 10–20% in NPSLE.^4^

Kidney disease, immunosuppression, or autoimmune disorders are frequently cited among the causes of PRES. There is more than one factor in the presented case, and the purpose of presenting this case is to compare the difference between Neuropsychiatric SLE and PRES in a patient with SLE and to highlight the factors that trigger the development of PRES in SLE.

## CASE

**Figure 1.**
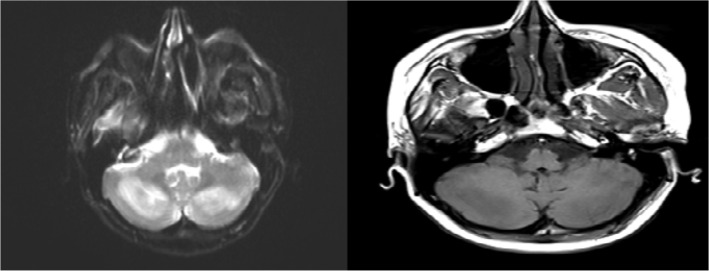
T2/FLAIR image of brain MRI. A 25-year-old female with systemic lupus erythematosus, presenting seizures after hypertension. Hyperintense signal changes in T2/FLAIR sequences in the left posterior frontal lobe, bilateral occipital, posterior parietal lobes, basal ganglia, and cerebellar hemispheres.

A 25-year-old female patient was admitted to the rheumatology outpatient clinic with complaints of malar redness on the face, pain in the wrist and fingers, swelling, hair loss, sores in the mouth, and low back pain about seven years ago. She was diagnosed with SLE and started on methylprednisolone and hydroxychloroquine, and the patient was followed up regularly. Proteinuria and haematuria were detected in the control examination of the patient, whose complaints regressed in the follow-up. A kidney biopsy was performed with the preliminary diagnosis of lupus nephritis. Class IV Lupus nephritis (LN) was found in the patient. The patient with Class IV LN was given two courses of rituximab treatment because she did not want cyclophosphamide treatment. She was followed up with azathioprine, methylprednisolone, hydroxychloroquine, and angiotensin-converting enzyme inhibitors. The patient, who was followed up with methylprednisolone, hydroxychloroquine, and furosemide the last year, was applied to our outpatient nephrology clinic with complaints of swelling on the feet and face. On physical examination, two positive pretibial oedemas were in both lower extremities. In the respiratory examination, thin rales were heard while listening to both lungs’ bases. Other system examinations were normal. Her vital signs were blood pressure: 170/80 mm/Hg SO2: 97, heart rate: 111/min, and fever: 36.4 degrees. Laboratory results of the case at the time of admission are given in **Table 1**. Since the patient’s eGFR level was below 10 ml/min and when the current clinical and laboratory status was evaluated, the patient was taken to emergency haemodialysis treatment because of accompanying hypervolemia findings. The patient was evaluated as chronic kidney disease as a result of laboratory and imaging results. The hypertensive patient had a generalised tonic-clonic seizure on our clinic’s 13th day of follow-up. During the seizure, the patient had symptoms of continuous daytime sleepiness, difficulty understanding what was said, and slow response. The patient with oral aphthae also had accompanying myositis findings. There was no sign of arthritis. In his laboratory, the C3 level the low, and C4 (C3 refrence:0.9–1.18 g/L - C4 reference 0.1–0.4 g/L) was normal, and lupus anticoagulant 34 sec (31–44 sec) was detected. The antiphospholipid antibody was negative. There was a telescopic urine finding in the complete urinalysis. The patient’s SLEDAI score was 44. Brain imaging (CT, MRI, venography) revealed hyperintense signal changes in T2/FLAIR sequences in the left posterior frontal lobe, bilateral occipital, posterior parietal lobes, basal ganglia, and cerebellar hemispheres (**Figure 1**). In the indicated areas, faint diffusion restriction areas were seen. A diagnosis of PRES syndrome was considered for the patient. EEG examination was evaluated as normal. PRES was deemed in the foreground, and levetiracetam 2x500 mg/day acetylsalicylic acid 100 mg/day started, and strict blood pressure monitoring and avoidance of hypertension were recommended. Since the patient required close vital follow-up, he was in the intensive care unit. She had a generalised tonic-clonic seizure in the intensive care unit for the second time. After a twoday intensive care follow-up, her blood pressure was controlled with antihypertensives, and she did not have recurrent seizures during her follow-up. The patient was followed up in the nephrology service. The patient, who did not develop any additional problems in the follow-up, was taken to the routine haemodialysis program and discharged.

**Table 1.** Laboratory values at the time of application.

**Parameters**	**Value**	REFERENCES
Urea mg/dl	334	16,6–48,5
Creatinin mg/dl	5,53	0,7–1,2
eGFR (MDRD) ml/min	9,98	
Sodium mmol/L	143	136–145
Potassium mmol/L	5,55	3,5–5,1
Phosphorus mg/dl	10,03	2,5–4,5
WBC 10^ 3/ul	10,13	4–10
Hemoglobin g/dl	10,1	12,1–17,2
Platelet Count 10^ 3/ul	491	150–400
Parathormone ng/l	306	15–65
Urine protein mg/24h	2700	<140
Urine albumin mg/24h	2200	<30
Ph (venous)	7,29	7,35–7,45
Pco2 mm/hg	27	35–45
Po2 mm/hg	58	75–100
HCO3 mmol/L	13,2	24–26

## DISCUSSION

SLE is a chronic autoimmune disease characterised by the production of autoantibodies against nuclear and cytoplasmic antigens, with many different clinical courses that can affect many other organs. In SLE patients, joint involvement, gastrointestinal, pulmonary, cardiovascular, hematological, ocular, skin, and peripheral and central nervous system involvement can be seen. The most common neurological manifestations are seizures and cerebrovascular events.^5^

The risk of death due to cere-bro-cardiovascular disease, infections, and kidney diseases in patients with SLE has increased three times compared to the general population. The main target of treatment; is to minimise comorbidities and drug toxicity by suppressing disease activity, preventing organ damage, and increasing survival. Corticosteroids, hydroxychloroquine, methotrexate, and nonsteroidal anti-inflammatory drugs (NSAIDs) are used in the treatment. As the symptoms progress, immunosuppressive cytotoxic agents are added to the treatment. In these patients, blood pressure control should be well maintained. Angiotensin-converting enzyme blockers or angiotensin receptor blockers are primarily preferred in antihypertensive therapy. These two groups of drugs delay the progression of lupus nephritis by maintaining the normotensive process and reducing proteinuria.^6^

### Posterior reversible encephalopathy syndrome

As in our patient, it can manifest only with generalised tonic-clonic seizures. Cerebral involvement due to bilateral posterior lobe infarcts, cerebral venous thrombosis, hypertensive encephalopathy, encephalitis, hypoglycemia, and hyponatremia should be considered in the differential diagnosis of PRES. In our case, venous thrombosis, infarction, ischemia conditions, infection, hypoglycemia, and hyponatremia were excluded with laboratory parameters. The formation mechanism of PRES is endothelial dysfunction caused by increased cerebral perfusion pressure secondary to hypertension. As a result, vasogenic oedema occurs. On imaging, vasogenic oedema mainly occurs in the posterior brain region, as in our patient. The greater involvement of the posterior hind brain is probably because arterial sympathetic innervation is maximal in the anterior circulation and decreases progressively posteriorly. Therefore, the occipital lobes and other hindbrain regions are at relatively higher risk for vasogenic oedema.^7^

PRES is frequently associated with acute and chronic kidney diseases. If kidney transplant, immunosuppressive drug use, hypertension (HT), and autoimmune diseases are present accompanying kidney disease, its incidence increases even more. Although hypertension has an important place in the pathophysiology of PRES, it has been shown that uremia, chronic kidney disease, and dialysis treatments also cause PRES. It has been shown that 50% of patients with PRES have HT, and 38% have chronic kidney damage. PRES should be considered in patients with acute onset neurological symptoms with uremia. Although the association of PRES and kidney damage is frequently mentioned in the literature, a study conducted in Ireland found PRES in 0.84% of the patients with the end-stage renal disease when the 10-year data were scanned retrospectively. They commented that three of these five patients developed during the first weeks of haemodialysis treatment, possibly secondary to changes in mean arterial blood pressure due to fluctuations in dry weight due to ultrafiltration. In the other two patients, it was stated that PRES developed three months after haemodialysis treatment.^8^ It has been suggested that pre-existing brain damage, mainly silent white matter lesions, predispose to PRES in patients with chronic kidney disease.^9^ In the case we presented, many factors, such as immunosuppressive use, HT, chronic kidney damage, and haemodialysis treatment would trigger the development of PRES. Although fluctuations in blood pressure were influential in the development of haemodialysis treatment in the initial period, the metabolic effects of uremia essentially paved the way for the development of PRES.

Most patients present with an acute elevation in blood pressure (BP); in these situations, antihypertensives targeting the same BP targets should be initiated as in other hypertensive emergencies. Seizures should be treated using rapidly loading IV drugs such as benzodiazepines. Second-line therapy includes antiepileptic drugs such as phenytoin or fosphenytoin. Discontinuation of the antiepileptic drug should be considered after the underlying triggers are controlled. Reversible damage has reported complete healing in 64–80% of cases, on average within 2–8 days, but it may take several weeks for full recovery to show. Mortality ranges from 4.8 to 16%. Deaths can be attributed to PRES due to status epilepticus, haemorrhagic complications, or herniation. Therefore, although PRES is considered a benign clinical entity, potential complications and long-term effects should be regarded based on the clinical characteristics of each patient.^10^

Among the autoimmune diseases, the disease most frequently associated with PRES is SLE. In addition, other PRES-related autoimmune diseases include systemic sclerosis, Henoch-Schönlein purpura, antineutrophil cytoplasm antibody-associated vasculitis, and rheumatoid arthritis. Neurological signs or symptoms can be detected in half of the patients with SLE. NPSLE should be considered when psychiatric and neurological symptoms develop in patients with SLE. Although NPSLE is seen in the early onset of SLE, it has been tried to be explained by two pathophysiological mechanisms, autoimmune neuroinflammatory and ischemic. The prevalence of NPSLE, which can affect the central and peripheral nervous system, was reported as 44.5% and 17.6% in retrospective studies. American College of Rheumatology tried to define it with central nervous system syndromes in NPSLE 7 peripheral 12, and PRES was not added to these. Diagnosis is difficult because there are no specific markers or laboratory findings.^11–13^ While autoantibodies anti-ribosomal-P, anti-NR2 in CSF fluid, and anti-neuronal antibodies are helpful, brain Magnetic Resonance imaging is the gold standard. Antihypertensives, anti-epileptic, anxiolytic, and anti-psychotics can be given in treating patients who develop NPSLE. Metabolic irregularities must be corrected. Anticoagulants with positive antiphospholipid antibodies should be added to antiaggregant therapy in ischemic NPSLE. Immunosuppressive therapies that have shown positive benefits are oral prednisolone and intravenous cyclophosphamide. The frequency of seizures decreased in those using hydroxychloroquine. Since most patients with NPSLE have ischemic and autoinflammatory mechanisms, combining the abovementioned treatments is required.^14–15^

Among the autoimmune diseases, the disease most frequently associated with PRESS is SLE. In addition, other PRES-related autoimmune diseases include systemic sclerosis, Henoch-Schönlein purpura, antineutrophil cytoplasmic antibody-associated vasculitis, and rheumatoid arthritis. Neurological signs or symptoms can be detected in half of SLE patients. Although 19 neuro-psychiatric syndromes have been identified during SLE, PRES is not one of them. Lupus nephritis, hypertension, high activity scores according to the Systemic Lupus Erythematosus Activity Index (SLEDAI), and recently immunosuppressive drug use has been identified as the most relevant risk factors for the SLE/PRES association. Although the frequency rates in the literature differ between studies, epileptic seizures have been reported at 7–16% in adult SLE cases and 51% in children.^16^ Common neuropsychiatric lupus (NPSLE) symptoms are headache, mood disorders, seizures, cognitive impairment, and cerebrovascular diseases. Although generalised tonic-clonic seizures are the most common type in NPSLE, superficial partial, complex partial, and rare status epilepticus can also be reported. Among the imaging methods, magnetic resonance imaging (MRI) is essential in diagnosing NPSLE with clinical findings. The most common MRI finding is small, punctuated, sub-cortical white matter foci, reported at a rate of 15–60%. It has been suggested that diffuse vascular changes in most patients with SLE and early atherosclerosis lead to cerebral hypoperfusion, resulting in this vasculitic appearance.

Lymphopenia is a common finding in SLE patients, which may further amplify IFN-induced endothelial disruption. Enhanced systemic endothelial activation, leukocyte leakage, and vasoconstriction, alone or in combination, can cause abnormal cerebral circulation. This pro-inflammatory and cytotoxic environment found in SLE may explain why PRES can occur without severe hypertension, as described in a subset of SLE-associated PRES cases (up to 20-30%).^17^

## CONCLUSION

Causes associated with PRES include infections, kidney disease, preeclampsia, cytotoxic drugs, and autoimmune diseases. SLE is the disease with the most evident among autoimmune diseases. SLE activity, hypertension, hematological and renal diseases, lymphopenia dyslipidemia, and immunosuppressive treatments are the causes that increase the risk of PRESS in SLE. In our case, the presence of SLE, hypertension, kidney damage requiring renal replacement therapy, and immunosuppressive therapy may have triggered the formation of PRESS. Therefore, in SLE patients with the aforementioned risk factor, one should be alert in terms of PRESS and must be differentiated from NPSLE.

## ABBREVIATIONS

BP: Blood pressure;
CT: Computed tomography;
EEG: Electroencephalography;
eGFR: Estimated/Glomerular filtration rate;
FLAIR: Fluid Attenuated Inversion Recovery;
HT: Hypertension;
IFN: Interferon;
IV: Intravenous;
LN: Lupus Nephritis;
MDRD: Modification of Diet in Renal Disease;
MRI: Magnetic resonance imaging;
NPSLE: Neuropsychiatric Lupus;
NSAID: Nonsteroidal anti-inflammatory drug;
PRES: Posterior reversible encephalopathy syndrome;
SLEDAI: Systemic Lupus Erythematosus Activity Index
